# Adoption of electronic patient-reported outcomes in cancer clinical practice: the point of view of Italian patients

**DOI:** 10.1016/j.esmorw.2024.100025

**Published:** 2024-02-19

**Authors:** A.A. Valsecchi, V. Battista, S. Terzolo, R. Dionisio, G. Lacidogna, D. Marino, V. Quarà, E. Sperti, V. Tuninetti, F. Vignani, C. Zichi, V.E. Bounous, G. Valabrega, A. Ferrero, N. Biglia, M. Di Maio

**Affiliations:** 1 Department of Oncology, University of Turin, Ordine Mauriziano Hospital, Turin, Italy; 2 Medical Oncology, Ordine Mauriziano—Umberto I Hospital, Turin, Italy; 3 Gynecology and Obstetrics Unit, Ordine Mauriziano—Umberto I Hospital, Department of Surgical Sciences, University of Turin, Turin, Italy

**Keywords:** patient-reported outcomes, quality of life, patient-reported outcome measures, electronic patient-reported outcome measures, cancer, clinical practice

## Abstract

**Background:**

In 2022, the European Society for Medical Oncology (ESMO) guideline recommended the adoption of electronic patient-reported outcomes measures (ePROMs) in routine clinical practice for patients with cancer. The aim of this survey is to identify the critical issues that would arise in the implementation of ePROMs in daily oncology clinical practice from patients’ view.

**Materials and methods:**

From March to April 2023, outpatients with cancer treated at Mauriziano Hospital in Turin (Italy) filled in a paper questionnaire. Seven questions dealt with the respondents’ characteristics, eight questions with the satisfaction about the current method used to collect information about patients’ symptoms and toxicities, and the opinion about several potentially critical issues for the implementation of ePROMs.

**Results:**

Two hundred and twenty patients completed the survey. In current clinical practice, symptoms are mostly collected through verbal questions and paper-based questionnaires. Seventy-two percent of patients were satisfied with these methods; 82% were in favor of using ePROMs. Most patients were not concerned about privacy (82%), while a minority was concerned about possible lack of interest by clinicians in the symptoms digitally reported (14%) and the possible negative impact on doctor–patient relationship (39%). Seventy-seven percent of respondents declared to be familiar with technological tools and 83% were confident with the availability of internet connection.

**Conclusions:**

In our center, most patients are satisfied with current methods of symptom monitoring and would also be in favor of the introduction of ePROMs. However, several aspects still need to be addressed for universal implementation of ePROMs in routine practice.

## Highlights

• In 2022, the ESMO published a guideline on the role of PROMs in the continuum of cancer care.
• The implementation of electronic patient-reported outcomes (ePROs) in routine clinical practice implies many challenges.
• This survey among Italian patients shows that a high proportion of patients are in favor of ePROs.
• The survey emphasizes that several issues need to be addressed.

## Introduction

Patient-reported outcomes (PROs) are defined as ‘any report of the status of a patient’s health condition that comes directly from the patient, without interpretation of the patient’s response by a clinician or anyone else’.^1^^,^^2^ Recently, the European Society for Medical Oncology (ESMO) published the first clinical practice guideline about the role of patient-reported outcome measures (PROMs) in the continuum of cancer clinical care, highlighting the importance of symptom monitoring via PROs.^1^

In fact, particularly in oncology, it is essential to manage patient’s symptoms properly and promptly (whether they are related to the disease, or to the toxicity of treatments) through optimal supportive care, in order to improve patients’ quality of life (QoL).^3^ To ensure this, it is essential to avoid under-detection and under-reporting of symptoms and underestimation of their severity. Symptom monitoring via PROs represents an evidence-based approach that responds to this need, overcoming the lack of concordance between symptom recognition by clinicians and patient self-reporting.^4^, ^5^, ^6^

To report and collect PROs, dedicated tools are available, called PROMs, typically questionnaires or standardized interview schedules, that the clinician can provide to the patient to better assess his/her symptoms and treatment’s adverse events (e.g. pain, nausea, vomiting, constipation, diarrhea, dyspnea, insomnia, depression, and physical function). In daily clinical practice, PROMs can promote communication between patients and clinicians, improving clinical management and guaranteeing optimal care quality.^7^^,^^8^

Electronic patient-reported outcome measures (ePROMs) are electronic instruments for the collection of PROs. The use of digital tools for administering PROMs to patients with cancer and communicating this information back to their clinicians has been recently recommended in routine clinical care, because it has been shown to improve satisfaction, treatment adherence, symptom control, physical function, QoL, adherence to treatment, reduction in emergency room and hospital admissions, and survival.^9^, ^10^, ^11^, ^12^ In fact, results of several recent randomized trials support the implementation of symptom monitoring in the routine workflow of medical oncology centers, with a digital solution.^10^^,^^11^^,^^13^, ^14^, ^15^ In addition, recently, Meirte et al. carried out a systematic meta-analysis of literature to provide an objective and comprehensive overview of the benefits and disadvantages of the digital collection of qualitative ePROMs: they seem to be preferred over paper-based methods, improve data quality, result in similar or faster completion time, decrease costs, and facilitate clinical decision making and symptom management.^10^

Unfortunately, despite the evidence cited above, to date, in Italy, the use of PROMs (especially ePROMs) is not yet part of routine clinical practice in the majority of oncology clinics. Information about the clinical usefulness, experience, usability, and perception and acceptability of these tools by clinicians and patients is still limited.

In some Italian centers, such as our hospital (Mauriziano Hospital, Turin), paper questionnaires have been introduced to collect PROs in routine practice.^3^ Thus, the aim of this survey is to evaluate the point of view of patients with cancer, who represent the end users of these digital tools, both in terms of satisfaction with current methods used to collecting symptoms and toxicities, and in terms of the critical issues that would arise in the potential future implementation of ePROMs in daily clinical practice.

## Materials and methods

The questionnaire used for this investigation consisted of a self-administered, multiple-choice paper questionnaire. It was developed by two authors, VB and MDM, specifically for this survey.

Thereafter, survey participants were recruited among the patients with appointments between 01 March 2023 and 30 April 2023, at the oncological day hospital at Mauriziano Hospital (Turin, Italy). The day hospital includes both patients treated by the Medical Oncology division and by the Gynecologic Oncology division. Overall, 220 patients fulfilled the questionnaire. Inclusion criteria were: patients over 18 years old; patients undergoing intravenous or oral cancer therapy; and Eastern Cooperative Oncology Group performance status (PS) 0-2. The criteria are deliberately broad in order to investigate the opinion of patients with heterogeneous characteristics, representative of those treated in clinical practice.

The answers to the questions were provided on a voluntary basis and all replies were anonymized. Approval for the anonymous distribution of the questionnaires was obtained from the School of Nursing (being the thesis work of VB) and from the Mauriziano Hospital Health Management.

Approval from the Ethical Committee was waived, due to the completely anonymous collection of the questionnaire (without any link to the clinical information of patients).

At the first access in our service, all patients had signed a written consent for the treatment of personal data, in anonymous format.

VB presented and explained to each patient the purpose of data collection and the questionnaire setting, helping patients to fill in if there were doubts.

The questionnaire, in Italian language, consisted of two parts. The first part, made by seven questions, was focused on the characteristics of the patients, while the second part, made by eight questions, dealt with the evaluation of the satisfaction of the current method used to collect information about symptoms and toxicities and the detection of any critical issues for the implementation of ePROMs in daily clinical practice.

In the second part, the first two questions were focused on the current method of collecting information about symptoms and toxicity (question 1) and patient satisfaction with that method (question 2). The third question asked about the willingness to use an electronic device (e.g. app on mobile phone, computer, or tablet) to assess treatment side-effects and symptoms experienced during oncology treatment. For the last five questions (from 4 to 8), the patient had to indicate how much she/he agreed with the proposed statement, with five levels of agreement (from strongly disagree, 0, to strongly agree, 4). The full questionnaire, translated in English language, is reported in Supplementary Table S1, available at https://doi.org/10.1016/j.esmorw.2024.100025. Our study questionnaire has not been previously utilized or published. The questions were chosen based on the review of existing publications.^10^

### Statistical analysis

Quantitative data were summarized by median, interquartile range, minimum, and maximum. Associations between categories were analyzed using the chi-squared test or linear-by-linear test for ordinal variables, as appropriate. Differences in continuous variables were assessed with the Wilcoxon Mann–Whitney test. All statistical tests were two-tailed and *P* values <0.05 were considered statistically significant. Because of the exploratory nature of the analysis, adjustment for multiple comparisons was not carried out. Analyses were carried out with IBM SPSS for Windows, version 28.0.1.0 (IBM Corp., Armonk, NY).

## Results

Two hundred and twenty oncological patients accepted to fill in the questionnaire.

Patients were heterogeneous in terms of primary tumors: the most common were breast cancer, gastrointestinal, urological, lung, and gynecological cancers. Both patients treated for metastatic disease and those with early-stage tumors receiving neoadjuvant or adjuvant treatment were included.

Females were 66%, and males, 34%. As for age, 42% of patients were younger than 60, 35% were between 61 and 70, and 23% were older than 70 years. Out of respondents, 49% were retired, 21% private employees, 11% private practitioners, 10% unemployed, and 9% public employees. Sixty-five percent of patients had high level of education, while 35% of them had low level of education. Most patients came to the hospital on a frequent basis (in detail: 60% every 2 weeks or more frequently, and 40% every 3 weeks or less frequently). The distances between hospital and home were distributed evenly among patients. In particular, in 48% of cases the distance was <10 km, and in 52% of cases >10 km. For a large majority of patients (79%), however, no difficulty was reported in reaching the hospital from home. Detailed respondents’ characteristics are reported in Table 1.

**Table 1:** Respondents’ characteristics

	Number/total number of respondents (%)
Sex	
Male	74/220 (34)
Female	146/220 (66)
Age, years	
<60	93/219 (42)
61-70	76/219 (35)
>70	50/219 (23)
Working status	
Public employee	18/211 (9)
Private employee	45/211 (21)
Private practitioner	23/211 (11)
Retired	104/211 (49)
Unemployed	21/211 (10)
Level of education	
None	1/217 (1)
Primary school	11/217 (5)
Secondary school first grade	64/217 (29)
Secondary school second grade	99/217 (46)
University	42/217 (19)
Timing schedule of oncological therapy	
Every week	57/215 (27)
Every 2 weeks	72/215 (33)
Every 3 weeks	75/215 (35)
Every 4 weeks	7/215 (3)
Every 6 weeks	4/215 (2)
Distance between hospital and home, km	
<5	49/216 (23)
5-10	54/216 (25)
10-20	40/216 (19)
>20	73/216 (33)
Difficulty coming to the scheduled visits	
Strongly disagree	157/199 (79)
Disagree	26/199 (13)
Uncertain	13/199 (7)
Agree	0/199 (0)
Strongly agree	3/199 (1)

According to patients’ responses, information about symptoms and toxicities were routinely collected in several ways (more than one answer was allowed): in most cases through questions asked by the clinician during the oncological visit (200/220) and, in descending order, through paper-based questionnaires (PROMs, 138/220), questions from a health care professional during the administration of therapy (109/220) or during collection of blood samples (84/220) or during the administration of therapy (4/220).

Most patients declared to be satisfied with the current method of symptom monitoring: in fact, 71.4% of patients rated satisfaction ≥8 on a scale from 1 to 10.

**Figure 1:**
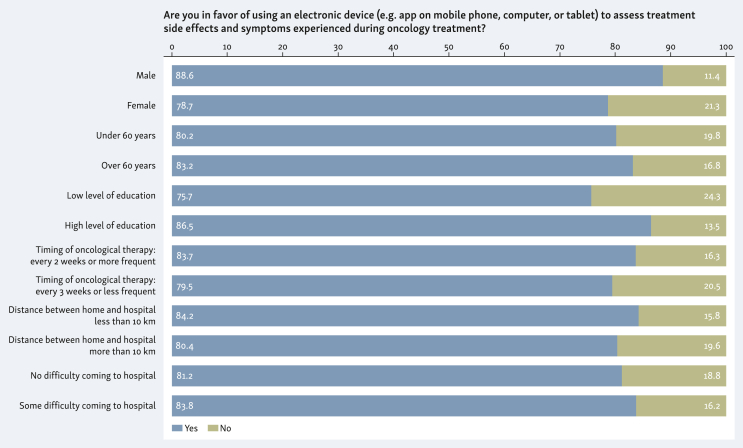
Response to question “Would you be in favor of using an electronic tool (e.g. app on your mobile phone, or on your computer or tablet) for the evaluation of patient-reported outcomes (PROs) (side-effects, toxicity, and any symptoms you may have during treatment)?” in the whole series of patients and in different subgroups.

Notably, 82% of patients were in favor of using an electronic device (e.g. app on mobile phone, computer, or tablet) to assess treatment side-effects and symptoms experienced during treatment (Figure 1). The proportion of patients in favor of using ePROs was similar across different subgroups.

**Figure 2:**
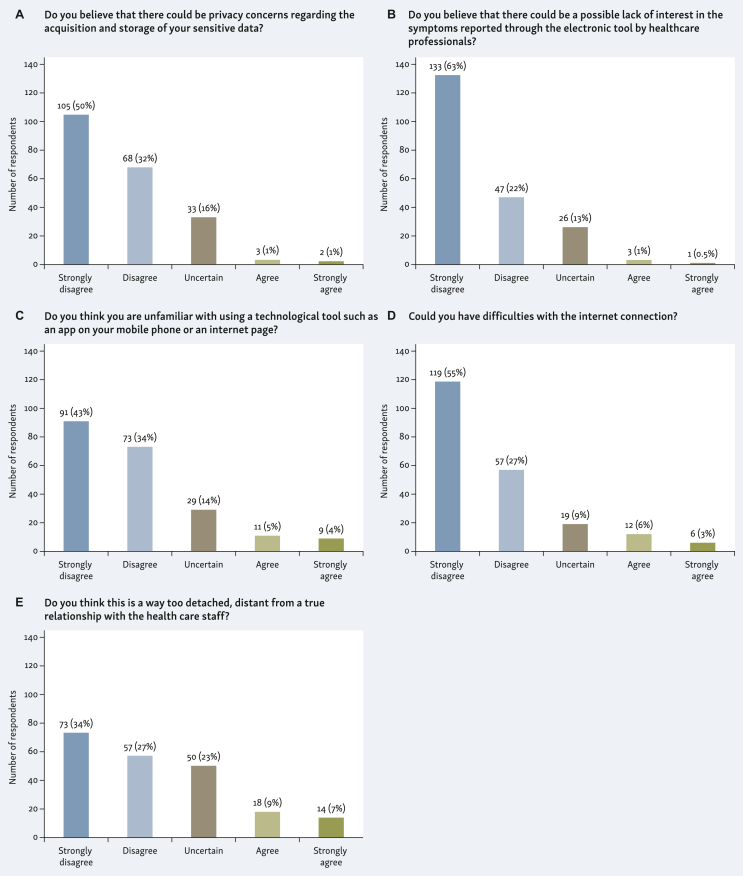
(A) Response to question “Do you believe that there could be privacy concerns regarding the acquisition and storage of your sensitive data?”; (B) Response to question “Do you believe that there could be a possible lack of interest in the symptoms reported through the electronic tool by health care professionals?”; (C) Responses to question “Do you think you are unfamiliar with using a technological tool such as an app on your mobile phone or an internet page?”; (D) Responses to question “Could you have difficulties with the internet connection?”; (E) Responses to question “Do you think this is a way too detached, distant from a true relationship with the health care staff?”.

Most patients (82%) declared that, with the use of ePROs, there would be no (50%) or few (32%) privacy concerns related to the acquisition and storage of sensitive personal data (Figure 2A). Among the remaining patients, the proportion of those who feared there might be some problems (agreement level 3 or 4 or 5) related to this topic was significantly higher among women (23% versus 9%, *P* = 0.015) (Supplementary Table S2, available at https://doi.org/10.1016/j.esmorw.2024.100025).

Most respondents (85%) did not believe (63%) or believed little (22%) that there could be a possible lack of interest by health care professionals in the symptoms reported through digital tools (Figure 2B). The proportion of those who were worried about this risk (agreement level 3 or 4 or 5) was higher among women (17% versus 9%, *P* = 0.048) and among people with a higher level of education (17% versus 9%, *P* = 0.004) (Supplementary Table S3, available at https://doi.org/10.1016/j.esmorw.2024.100025).

Most patients (77%) confirmed they were familiar (43%) or just a little unfamiliar (34%) with using a technological tool such as a mobile app or an internet website (Figure 2C). Patients older than 60 years (26% versus 19%, *P* = 0.007) or patients with a low grade of education (38% versus 15%, *P* < 0.001) were those most likely to declare poor handling of computer devices (agreement level 3 or 4 or 5) (Supplementary Table S4, available at https://doi.org/10.1016/j.esmorw.2024.100025).

Most respondents (82%) declared to have no (55%) or only little difficulty (27%) with internet connection (Figure 2D). As for the previous question, those who complained higher difficulties (agreement level 3 or 4 or 5) were the patients older than 60 years (19% versus 16%, *P* = 0.016) and those with a low grade of education (27% versus 12%, *P* = 0.003) (Supplementary Table S5, available at https://doi.org/10.1016/j.esmorw.2024.100025).

Most patients (61%) were not concerned at all (34%) or just a little concerned (27%) that the implementation of technological tools was an approach too detached and distant from a ‘true’ relationship with the clinician (Figure 2E). The proportion of those believing that this approach could compromise the doctor–patient relationship (agreement level 3 or 4 or 5) was higher among women (44% versus 28%, *P* = 0.01) (Supplementary Table S6, available at https://doi.org/10.1016/j.esmorw.2024.100025).

## Discussion

Following the publication of ESMO guidelines about the role of PROs in cancer clinical care, every center should plan the implementation of ePROs in routine clinical practice. This survey explored the point of view of outpatients with cancer treated at Mauriziano Hospital, in Turin, Italy, about the implementation of ePROs in daily practice. In our center, since 2018, the use of paper-based PROs to collect information about symptoms and treatment-related toxicities has been routinely adopted in clinical practice, following a positive initial experience, which showed a significant benefit in terms of patients’ QoL, satisfaction, and a positive feedback from doctors and nurses.^3^

Indeed, paper-based tools allow a better communication between health care operators and patient during the visit, helping to reduce under-reporting of symptoms and toxicities, but these instruments do not allow a real-time flow of information and timely management of clinical problems. From this point of view, implementing digital tools for remote monitoring of patients’ symptoms is becoming a hot topic in the landscape of oncology.

Despite several studies and randomized trials having demonstrated that the use of ePROMs can improve QoL (and in some cases survival) of patients receiving cancer treatment or in follow-up, enhancing the communication with clinicians and patients’ satisfaction, information on the clinical applicability and on the user impressions about these systems is still limited.

A recent systematic review examined both the benefits and barriers of the digital collection of qualitative ePROMs, showing that, on one hand, these tools facilitate symptom management and patient–physician communication and are characterized by high patient preference and acceptance, low cost, and higher data quality and response rates; on the other hand, ePROMs have potential drawbacks, including privacy concerns, a potentially high initial financial investment, and risk of exclusion of certain populations.^10^

In this setting, our survey was conducted with the aim of exploring patients’ perspectives on the adoption of these digital tools in daily clinical practice. Most responses are consistent with the findings of the review cited previously.

To date, in most cases, respondents’ symptoms have been assessed by verbal questions asked by physicians during the visit or by paper questionnaires given to the patient, completed at home or in hospital before the visit and returned to the physician at the next visit. Most patients (72%) in our survey declared to be satisfied with this approach. In our initial experience, satisfaction with the use of paper-based questionnaires was very high: 92% of patients declared that the questionnaire was clear and easy to understand, 93% declared that it was useful to report symptoms and side-effects, and 88% thought that the questionnaire was a valid instrument to improve communication with the physician.^3^

Despite the high level of satisfaction with the method currently used in our hospital to record symptoms and toxicities, 82% of patients, regardless of sex, age, educational attainment, or distance between home and hospital, declared that they would be in favor of the use of an electronic device to assess side-effects and symptoms during oncology treatment.

Patients’ feedback shows that the impact of ePROMs on quality and value of care is positively perceived: most patients do not believe that implementation of this new approach could significantly interfere with the direct doctor–patient relationship (61%) or reduce the doctor’s interest in discussing their symptoms (86%). Of note, women and patients with a higher level of education are more worried about potential disadvantages in these issues. From these results it clearly emerges that digital tools must be perceived by patients as an added value in the treatment process and not as a substitute or a surrogate for the traditional relationship between doctor and patient, which is obviously considered crucial by the patients themselves. The physician must therefore well explain to patients the clinical relevance and the potential value of this new approach for their care.

Among the possible obstacles to ePROMs identified in the systematic review cited previously, there were aspects related to patient privacy and the possible exclusion of a subset of the population, poorly familiar with technology or with difficulties accessing the internet. Regarding the former point, most respondents (82%) in our survey were not significantly concerned about privacy. Regarding the latter point, despite the fact that most respondents report being familiar with the use of electronic devices (77%) and having no difficulties accessing the internet (83%), there is a minority of the population that is unwilling or unable to complete ePROMs due to their older age, computer ignorance, or low level of education. Thus, although our results are all in favor of ePROMs, we cannot ignore potential barriers that may exclude subgroups of the population from receiving the best possible health care. Possible solutions should be kept in mind when developing ePROMs, to optimize usability and enable all population of patients to optimally use the digital data collection tool. ESMO clinical practice guideline states that, when feasible, more than one mode of administration should be offered, to ensure that vulnerable populations are able to have access to a survey platform. In the absence of a digital solution, even paper-based administration of questionnaires could be considered useful.

Our survey has several limitations.

Firstly, the number of patients is small and does not allow a complete generalization of the results, although they are consistent with the results of similar experiences present in the literature on this topic. For example, in an exploratory study investigating through a questionnaire the use of ePROMs for follow-up after palliative radiotherapy, both patients and health care providers agree that ePROMs could improve systematic clinical follow-up.^16^ Similarly, another study exploring patient experiences of using the ePROMs service showed that they feel ePROMs use improved communication and increased the feeling of involvement with their care.^17^

Secondly, it is limited to a single center (with nearly all patients coming from the same city/geographical area) and results cannot be considered representative of the whole Italian population.

Thirdly, due to the case mix treated at our center, the study population was quite younger than the typical population of patients with cancer, and this could have underestimated the difficulties with technology, typically higher in older subjects.

Fourthly, the survey was limited to patients and did not explore the point of view of health care professionals, such as physicians and nurses, who, along with patients, are the main actors in the treatment pathway.

Fifthly, it was not explicitly required in the questionnaire how many times patients were normally asked PROs in different ways (verbal or paper). In our experience, this data collection was made on each visit verbally and giving a paper questionnaire, to be completed at home and returned at the next visit, indicating the symptoms experienced in the time elapsed.

Patients’ point of view is certainly crucial for the implementation of a tool validated by several clinical trials in clinical practice, but unfortunately, the practical implementation of ePROMs must also take into account other technical aspects: the need of financial resources; the acquisition and technical maintenance of the digital tool; the interface of the software with the electronic health record; and a quantitatively adequate health staff dedicated to read in real time the data transmitted by patients on the digital platform and to provide them with timely feedback. All these issues should be investigated and explored in depth, to allow an efficient and effective implementation of these new tools in daily clinical practice.

In conclusion, this study shows that efforts of introducing systematic assessment of symptoms and toxicities with paper questionnaires in the routine care of outpatients with cancer are worthwhile, because most patients are satisfied with current methods of symptom monitoring. However, they would also be in favor of the introduction of digital tools, despite some concerns from a minority of the respondents. Several aspects still need to be defined to make the use of ePROMs part of routine practice, but most patients seem to be well prepared for this change.
