# Supplementary material for: Adoption of electronic patient-reported outcomes in cancer clinical practice: the point of view of Italian patients
Source: ESMO Real World Data Digit Oncol. 2024 Feb 19;3:100025. doi: 10.1016/j.esmorw.2024.100025 (PMC12836719; doi:10.1016/j.esmorw.2024.100025)
Supplement: Supplementary Tables S1–S6 [file mmc1.docx]

**Supplementary material**

**Supplementary Table 1.** Questionnaire translated into English language

| **Part one** |  |
| --- | --- |
| **Question** | **Options** |
| 1. **Sex** |  |
|  | Male |
|  | Female |
|  | I prefer not to answer |
| 1. **Age** |  |
|  | *Free text* |
| 1. **Working status** |  |
|  | Public employee |
|  | Private employee |
|  | Private Practitioner |
|  | Pensioner |
|  | Unemployed |
| 1. **Graduation** |  |
|  | None |
|  | Primary school |
|  | Secondary school first grade |
|  | Secondary school second grade |
|  | University |
| 1. **Timing schedule of oncological therapy** |  |
|  | Every week |
|  | Every two weeks |
|  | Every three weeks |
|  | Every 28 days |
|  | Every six weeks |
| 1. **Distance between hospital and home** |  |
|  | Less than 5 km |
|  | Between 5 to 10 km |
|  | Between 10 to 20 km |
|  | More than 20 km |
| 1. **Difficulty coming to the scheduled visits** |  |
|  | Strongly disagree |
|  | Disagree |
|  | Uncertain |
|  | Agree |
|  | Strongly agree |
| **Part two** |  |
| 1. **Now how are the toxicity and symptoms you have after the therapy evaluated?** |  |
|  | **Paper questionnaires** |
|  | **Questions from a healthcare professional during blood sampling** |
|  | **Questions from a doctor during the medical visit** |
| 1. **How satisfied is from 1 to 10 with the current method of reporting toxicity and symptoms?** |  |
|  | *Insert number from 1 to 10* |
| 1. **Would you be in favor of using an electronic tool (e.g. app on your mobile phone, or on your computer or tablet) for the evaluation of patient-reported outcomes (PROs) (side effects, toxicity, and any symptoms you may have during treatment)?** |  |
|  | Yes |
|  | No |
| 1. **Do you believe that there would be privacy concerns regarding the acquisition and storage of your sensitive data?** |  |
|  | Strongly disagree |
|  | Disagree |
|  | Uncertain |
|  | Agree |
|  | Strongly agree |
| 1. **Do you believe that there could be a possible lack of interest in the symptoms reported through the electronic tool by healthcare professionals?** |  |
|  | Strongly disagree |
|  | Disagree |
|  | Uncertain |
|  | Agree |
|  | Strongly agree |
| 1. **Do you think you are unfamiliar with using a technological tool such as an app on your mobile phone or an internet page?** |  |
|  | Strongly disagree |
|  | Disagree |
|  | Uncertain |
|  | Agree |
|  | Strongly agree |
| 1. **Could you have difficulties with the internet connection?** |  |
|  | Strongly disagree |
|  | Disagree |
|  | Uncertain |
|  | Agree |
|  | Strongly agree |
| 1. **Do you think this is a way too detached, distant from a true relationship with the health care staff?** |  |
|  | Strongly disagree |
|  | Disagree |
|  | Uncertain |
|  | Agree |
|  | Strongly agree |

**Supplementary Table 2.** D**etailed subgroup analysis for Question n. 4, Part 2: “**Response to question “Do you believe that there could be privacy concerns regarding the acquisition and storage of your sensitive data?**”.**

|  | **Strongly disagree** | **Disagree** | **Uncertain** | **Agree** | **Strongly agree** | **P value** |
| --- | --- | --- | --- | --- | --- | --- |
| **All patients** | 105 (49.8%) | 68 (32.2%) | 33 (15.6%) | 3 (1.4%) | 2  (0.9%) |  |
| **Sex** |  |  |  |  |  | 0.15 |
| Male | 40 (57.1%) | 24 (34.3%) | 6  (8.6%) | 0  (0%) | 0  (0%) |  |
| Female | 65 (46.1%) | 44 (31.2%) | 27 (19.1%) | 3  (2.1%) | 2  (1.4%) |  |
| **Age** |  |  |  |  |  | 0.87 |
| Under 60 years | 41  (46.6%) | 31  (35.2%) | 15  (17%) | 1  (1.1%) | 0  (0%) |  |
| Over 60 years | 64  (52%) | 37  (30.1%) | 18  (14.6%) | 2  (1.6%) | 2  (1.6%) |  |
| **Level of education** |  |  |  |  |  | 0.20 |
| Low | 33  (45.8%) | 22  (30.6%) | 15  (20.8%) | 1  (1.4%) | 1  (1.4%) |  |
| High | 72  (51.8%) | 46  (33.1%) | 18  (12.9%) | 2  (1.4%) | 1  (0.7%) |  |
| **Timing of oncological therapy** |  |  |  |  |  | 0.11 |
| Every 2 weeks or more frequent | 66  (53.2%) | 39  (31.5%) | 17  (13.7%) | 2  (1.6%) | 0  (0%) |  |
| Every 3 weeks or less frequent | 39  (44.8%) | 29  (33.3%) | 16  (18.4%) | 1  (1.1%) | 2  (2.3%) |  |
| **Distance between home and**  **hospital** |  |  |  |  |  | 0.78 |
| Less than 10 km | 51  (20.5%) | 32  (31.7%) | 14  (13.9%) | 2  (2%) | 2  (2%) |  |
| More than 10 km | 54  (49.1%) | 36  (32.7%) | 19  (17.3%) | 1  (0.9%) | 0  (0%) |  |
| **Difficulty coming to hospital** |  |  |  |  |  | 0.06 |
| No | 83  (54.2%) | 43  (28.1%) | 26  (17%) | 0  (0%) | 1  (0.7%) |  |
| Some | 22  (37.9%) | 25  (43.1%) | 7  (12.1%) | 3  (5.2%) | 1  (1.7%) |  |

**Supplementary Table 3.** D**etailed subgroup analysis for Question n. 5, Part 2: “**Response to question “Do you believe that there could be a possible lack of interest in the symptoms reported through the electronic tool by healthcare professionals?**”.**

|  | **Strongly disagree** | **Disagree** | **Uncertain** | **Agree** | **Strongly agree** | **P value** |
| --- | --- | --- | --- | --- | --- | --- |
| **All patients** | 133  (63.3%) | 47  (22.4%) | 26  (12.4%) | 3  (1.4%) | 1  (0.5%) |  |
| **Sex** |  |  |  |  |  | **0.048** |
| Male | 50  (70.4%) | 15  (21.1%) | 6  (8.5%) | 0  (0%) | 0  (0%) |  |
| Female | 83  (59.7%) | 32  (23%) | 20  (14.4%) | 3  (2.2%) | 1  (0.7%) |  |
| **Age** |  |  |  |  |  | 0.84 |
| Under 60 years | 54  (62.8%) | 19  (22.1%) | 11  (12.8%) | 2  (2.3%) | 0  (0%) |  |
| Over 60 years | 79  (63.7%) | 28  (22.6%) | 15  (12.1%) | 1  (0.8%) | 1  (0.8%) |  |
| **Level of education** |  |  |  |  |  | **0.004** |
| Low | 54  (78.3%) | 9  (13%) | 6  (8.7%) | 0  (0%) | 0  (0%) |  |
| High | 79  (56%) | 38  (27%) | 20  (14.2%) | 3  (2.1%) | 1  (0.7%) |  |
| **Timing of oncological therapy** |  |  |  |  |  | 0.38 |
| Every 2 weeks or more frequent | 79  (64.8%) | 27  (22.1%) | 15  (12.3%) | 1  (0.8%) | 0  (0%) |  |
| Every 3 weeks or less frequent | 54  (61.4%) | 20  (22.7%) | 11  (12.5%) | 2  (2.3%) | 1  (1.1%) |  |
| **Distance between home and**  **Hospital** |  |  |  |  |  | 0.33 |
| Less than 10 km | 62  (62%) | 21  (21%) | 14  (14%) | 2  (2%) | 1  (1%) |  |
| More than 10 km | 71  (64.5%) | 26  (23.6%) | 12  (10.9%) | 1  (0.9%) | 0  (0%) |  |
| **Difficulty coming to hospital** |  |  |  |  |  | 0.12 |
| No | 102  (67.1%) | 31  (20.4%) | 16  (10.5%) | 2  (1.3%) | 1  (0.7%) |  |
| Some | 31  (53.4%) | 16  (27.6%) | 10  (17.2%) | 1  (1.7%) | 0  (0%) |  |

**Supplementary Table 4.** D**etailed subgroup analysis for Question n. 6, Part 2:** “Do you think you are unfamiliar with using a technological tool such as an app on your mobile phone or an internet page?”.

|  | **Strongly disagree** | **Disagree** | **Uncertain** | **Agree** | **Strongly agree** | **P value** |
| --- | --- | --- | --- | --- | --- | --- |
| **All patients** | 91  (42.7%) | 73  (34.3%) | 29  (13.6%) | 11  (5.2%) | 9  (4.2%) |  |
| **Sex** |  |  |  |  |  | 0.30 |
| Male | 34  (47.9%) | 23  (32.4%) | 8  (11.3%) | 4  (5.6%) | 2  (2.8%) |  |
| Female | 57  (40.1%) | 50  (35.2%) | 21  (14.8%) | 7  (4.9%) | 7  (4.9%) |  |
| **Age** |  |  |  |  |  | **0.007** |
| Under 60 years | 50  (56.8%) | 21  (23.9%) | 12  (13.6%) | 3  (3.4%) | 2  (2.3%) |  |
| Over 60 years | 41  (32.8%) | 52  (41.6%) | 17  (13.6%) | 8  (6.4%) | 7  (5.6%) |  |
| **Level of education** |  |  |  |  |  | **<0.001** |
| Low | 18  (24.7%) | 27  (37%) | 18  (24.7%) | 5  (6.8%) | 5  (6.8%) |  |
| High | 73  (52.1%) | 46  (32.9%) | 11  (7.9%) | 6  (4.3%) | 4  (2.9%) |  |
| **Timing of oncological therapy** |  |  |  |  |  | 0.26 |
| Every 2 weeks or more frequent | 52  (41.6%) | 42  (33.6%) | 16  (12.8%) | 8  (6.4%) | 7  (5.6%) |  |
| Every 3 weeks or less frequent | 39  (44.3%) | 31  (35.2%) | 13  (14.8%) | 3  (3.4%) | 2  (2.3%) |  |
| **Distance between home and**  **hospital** |  |  |  |  |  | 0.62 |
| Less than 10 km | 43  (43%) | 36  (36%) | 13  (13%) | 4  (4%) | 4  (4%) |  |
| More than 10 km | 48  (42.5%) | 37  (32.7%) | 16  (14.2%) | 7  (6.2%) | 5  (4.4%) |  |
| **Difficulty coming to hospital** |  |  |  |  |  | 0.17 |
| No | 70  (45.5%) | 51  (33.1%) | 20  (13%) | 8  (5.2%) | 5  (3.2%) |  |
| Some | 21  (35.6%) | 22  (37.3%) | 9  (15.3%) | 3  (5.1%) | 4  (6.8%) |  |

**Supplementary Table 5.** D**etailed subgroup analysis for Question n. 7, Part 2:** “Could you have difficulties with the internet connection?”.

|  | **Strongly disagree** | **Disagree** | **Uncertain** | **Agree** | **Strongly agree** | **P value** |
| --- | --- | --- | --- | --- | --- | --- |
| **All patients** | 119  (55.9%) | 57  (26.8%) | 19  (8.9%) | 12  (5.6%) | 6  (2.8%) |  |
| **Sex** |  |  |  |  |  | 0.16 |
| Male | 40  (57.1%) | 22  (31.4%) | 6  (8.6%) | 1  (1.4%) | 1  (1.4%) |  |
| Female | 79  (55.2%) | 35  (24.5%) | 13  (9.1%) | 11  (7.7%) | 5  (3.5%) |  |
| **Age** |  |  |  |  |  | **0.016** |
| Under 60 years | 60  (67.4%) | 15  (16.9%) | 10  (11.2%) | 4  (4.5%) | 0  (0%) |  |
| Over 60 years59 | 59  (47.6%) | 42  (33.9%) | 9  (7.3%) | 8  (6.5%) | 6  (4.8%) |  |
| **Level of education** |  |  |  |  |  | **0.003** |
| Low | 34  (46.6%) | 19  (26%) | 10  (13.7%) | 5  (6.8%) | 5  (6.8%) |  |
| High | 85  (60.7%) | 38  (27.1%) | 9  (6.4%) | 7  (5%) | 1  (0.7%) |  |
| **Timing of oncological therapy** |  |  |  |  |  | 0.52 |
| Every 2 weeks or more frequent | 65  (51.6%) | 36  (28.6%) | 16  (12.7%) | 5  (4%) | 4  (3.2%) |  |
| Every 3 weeks or less frequent | 54  (62.1%) | 21  (24.1%) | 3  (3.4%) | 7  (8%) | 2  (2.3%) |  |
| **Distance between home and**  **hospital** |  |  |  |  |  | 0.10 |
| Less than 10 km | 57  (57%) | 27  (27%) | 10  (10%) | 3  (3%) | 3  (3%) |  |
| More than 10 km | 62  (54.9%) | 30  (26.5%) | 9  (8%) | 9  (8%) | 3  (2.7%) |  |
| **Difficulty coming to hospital** |  |  |  |  |  | 0.06 |
| No | 92  (59.7%) | 38  (24.7%) | 12  (7.8%) | 9  (5.8%) | 3  (1.9%) |  |
| Some | 27  (45.8%) | 19  (32.2%) | 7  (11.9%) | 3  (5.1%) | 3  (5.1%) |  |

**Supplementary Table 6.** D**etailed subgroup analysis for Question n. 8, Part 2:** “Do you think this is a way too detached, distant from a real relationship with the health care staff?”.

|  | **Strongly disagree** | **Disagree** | **Uncertain** | **Agree** | **Strongly agree** | **P value** |
| --- | --- | --- | --- | --- | --- | --- |
| **All patients** | 73  (34.4%) | 57  (26.9%) | 50  (23.6%) | 18  (8.5%) | 14  (6.6%) |  |
| **Sex** |  |  |  |  |  | **0.01** |
| Male | 29  (40.8%) | 22  (31%) | 16  (22.5%) | 2  (2.8%) | 2  (2.8%) |  |
| Female | 44  (31.2%) | 35  (24.8%) | 34  (24.1%) | 16  (11.3%) | 12  (8.5%) |  |
| **Age** |  |  |  |  |  | 0.25 |
| Under 60 years | 30  (33.7%) | 22  (24.7%) | 19  (21.3%) | 10  (11.2%) | 8  (9%) |  |
| Over 60 years | 43  (35%) | 35  (28.5%) | 31  (25.2%) | 8  (6.5%) | 6  (4.9%) |  |
| **Level of education** |  |  |  |  |  | 0.08 |
| Low | 31  (43.1%) | 16  (22.2%) | 18  (25%) | 4  (5.6%) | 3  (4.2%) |  |
| High | 42  (30%) | 41  (29.3%) | 32  (22.9%) | 14  (10%) | 11  (7.9%) |  |
| **Timing of oncological therapy** |  |  |  |  |  | 0.28 |
| Every 2 weeks or more frequent | 46  (36.8%) | 34  (27.2%) | 30  (24%) | 6  (4.8%) | 9  (7.2%) |  |
| Every 3 weeks or less frequent | 27  (31%) | 23  (26.4%) | 20  (23%) | 12  (13.8%) | 5  (5.7%) |  |
| **Distance between home and**  **hospital** |  |  |  |  |  | 0.98 |
| Less than 10 km | 34  (33.7%) | 26  (25.7%) | 27  (26.7%) | 9  (8.9%) | 5  (5%) |  |
| More than 10 km | 39  (35.1%) | 31  (27.9%) | 23  (20.7%) | 9  (8.1%) | 9  (8.1%) |  |
| **Difficulty coming to hospital** |  |  |  |  |  | 0.53 |
| No | 56  (36.4%) | 38  (24.7%) | 37  (24%) | 15  (9.7%) | 8  (5.2%) |  |
| Some | 17  (29.3%) | 19  (32.8%) | 13  (22.4%) | 3  (5.2%) | 6  (10.3%) |  |
